# Structure, luminescence and temperature sensing in rare earth doped glass ceramics containing NaY(WO_4_)_2_ nanocrystals

**DOI:** 10.1039/c8ra00190a

**Published:** 2018-02-19

**Authors:** Zeshang Zou, Ting Wu, Hao Lu, Yuyuan Tu, Shilong Zhao, Shichao Xie, Fang Han, Shiqing Xu

**Affiliations:** College of Materials Science and Engineering, China Jiliang University Hangzhou 310018 P. R. China Shilong_zhao@hotmail.com sxucjlu@hotmail.com

## Abstract

Novel rare earth doped glass ceramics containing NaY(WO_4_)_2_ nanocrystals were fabricated for the first time. The appearance of sharp diffraction peaks and well-resolved lattice fringes certifies the precipitation of NaY(WO_4_)_2_ nanocrystals with high crystallinity. After the crystallization process, significant changes in the photoluminescence emission spectra and fluorescence lifetime of Sm^3+^ ions are observed, which are ascribable to the enrichment of Sm^3+^ ions in the highly disordered NaY(WO_4_)_2_ nanocrystals. Under 980 nm excitation, characteristic green and red upconversion emission signals were detected and the enhanced upconversion luminescence of Er^3+^ ions in the glass ceramics was attributable to the incorporation into the low energy phonon NaY(WO_4_)_2_ nanocrystals. Based on the dependence of upconversion intensity on the excitation power, the upconversion mechanism of Er^3+^–Yb^3+^ ions was proposed. The temperature-dependent fluorescence intensity ratio (FIR) of the thermally-coupled ^2^H_11/2_ and ^4^S_3/2_ energy levels was determined at a low power density of 0.4125 W cm^−2^. The maximum temperature sensitivity is 146 × 10^−4^ K^−1^ at 523 K, which is mainly attributed to the highly disordered structure of NaY(WO_4_)_2_ nanocrystals and exhibits promising potential for optical temperature sensors.

## Abstract 2

A novel rare earth doped glass ceramic containing NaY(WO_4_)_2_ nanocrystals was fabricated and its temperature sensing properties were investigated.

## Graphical abstract

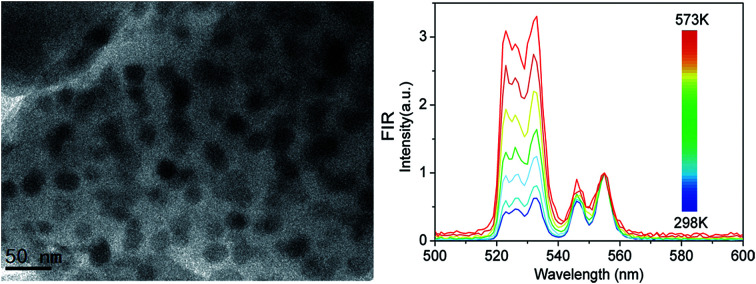

## Introduction

In recent years, upconversion luminescence of active ions has attracted wide interest owing to its promising applications in bioimaging, solar cells, white LEDs and temperature sensing.^1–4^ Especially, temperature sensors based on the FIR technique have garnered great attention due to their negligible electromagnetic interference, simple data processing and high sensitivity.^4–7^ Currently, the research of high-performance temperature sensors mainly focuses on the optimization of active ions and host matrix. Er^3+^ ion is the most extensively investigated because of its intense upconversion luminescence and appropriate bandgap (around 800 cm^−1^) between ^2^H_11/2_ and ^4^S_3/2_ energy levels, which exactly fall in the thermally-coupled energy range (200–2000 cm^−1^).^8^ In order to obtain stronger upconversion luminescence, Yb^3+^ is usually utilized as the preferred sensitizer for Er^3+^ due to its high absorption cross-section at 980 nm and efficient Yb^3+^ → Er^3+^ energy transfer efficiency.^9^ It is well known that the phonon energy and crystal structure of host matrix have an important role on the upconversion luminescence efficiency. A low phonon energy is very beneficial to enhance the upconversion efficiency of activators as a result of the reduction in the multi-phonon relaxation rate.^10^ Hexagonal NaYF_4_ is regarded as the most efficient upconversion host matrix due to its low phonon energy and multisite characteristic.^11^ The temperature sensing performance of Er^3+^ ions in hexagonal NaYF_4_ was studied and the maximum temperature sensitivity was 37 × 10^−4^ K^−1^ at 508 K.^12^ By now, various host materials have been investigated for optical thermometry, however, the influence of crystal structure on the temperature sensitivity is rarely investigated.^13^ Recently, three Judd–Ofelt parameters^14,15^ of Er^3+^ ions were calculated and used to analyze the role of host matrix from oxyfluoride glass to cubic NaYF_4_ glass ceramic on the temperature sensitivity. It was found that the maximum temperature sensitivity of Er^3+^ ions in the precursor glass (66 × 10^−4^ K^−1^ at 570 K) is twice more than the value in the cubic NaYF_4_ glass ceramic (24 × 10^−4^ K^−1^ at 540 K). Based on the structural change during the temperature-induced crystallization process, it is concluded that the more disordered the local environment of active ions, the better FIR and temperature sensing sensitivity. Thus, oxide glass is regarded as an ideal host matrix doped with Er^3+^ ions for optical thermometry due to its highly disordered local symmetry surroundings.^16^ Unfortunately, a low upconversion efficiency of active ions can only be obtained in oxide glasses due to their high phonon energy.

Rare earth doped double tungstate/molybdate phosphors have been extensively investigated due to their structural versatility, good thermal stability and low phonon energy. Recently, intense green upconversion luminescence was observed in the Er^3+^/Yb^3+^ codoped NaY(WO_4_)_2_ phosphors and its temperature sensing behaviour was analysed by the FIR technique.^17^ A maximum temperature sensing sensitivity about 112 × 10^−4^ K^−1^ was obtained at 515 K, which was about three times larger than that in the NaYF_4_ nanocrystals^12^ and exhibited promising potential for application on optical thermometry. Moreover, in order to assemble a practical optical fiber temperature sensing probe, the phosphor is usually adhered to the end face of optical fiber by organic resin (epoxy resin or silicone). Therefore, the operating temperature range, long-term reliability and lifetime of optical fiber temperature sensor are reduced dramatically. Glass can be easily drawn into glass fiber and conveniently spliced to standard silica fiber. The combination of oxide glass and tungstate/molybdate phosphor is expected to improve the performance of temperature sensors. Therefore, the key problem is to develop an appropriate glass composition, in which tungstate/molybdate nanocrystals could be successfully separated from the precursor glass and intense upconversion emission could be achieved.

Here, transparent glass ceramics containing NaY(WO_4_)_2_ nanocrystals have been successfully prepared by the optimization of glass composition and thermal treatment condition. Sm^3+^ ion usually acts as a spectroscopic probe to distinguish the local site symmetry around active ions in the host matrix. The spectral analysis of Sm^3+^ ions in the precursor glass and glass ceramics containing NaY(WO_4_)_2_ nanocrystals has been carried out and used to estimate their potential performance for optical temperature sensing. Successively, the temperature dependent upconversion emission spectra in Er^3+^/Yb^3+^ codoped glass and glass ceramics were systematically investigated. According to FIR technique, the influence of host matrix on the temperature sensitivity was discussed in details.

## Experimental

### Sample preparation

The glass samples were fabricated with molar composition of 62SiO_2_–16B_2_O_3_–11Na_2_O–7ZnO–3WO_3_–0.9Y_2_O_3_–0.1Sm_2_O_3_ and 62SiO_2_–16B_2_O_3_–11Na_2_O–7ZnO–3WO_3_–0.75Y_2_O_3_–0.05Er_2_O_3_–0.2 Yb_2_O_3_. Firstly, high purity raw materials SiO_2_, H_3_BO_3_, Na_2_CO_3_, ZnO, WO_3_, Y_2_O_3_, Sm_2_O_3_, Er_2_O_3_ and Yb_2_O_3_ were weighed accurately and mixed thoroughly. Then, the well-mixed raw materials were put into a covered Al_2_O_3_ crucible and were transferred to an electric furnace preheated to 1500 °C for about 30 min. Finally, the glass melt was quenched into a cold stainless-steel plate and rapidly transferred to a pre-heated muffle furnace. Transparent precursor glasses (PG) were obtained. PG samples were cut into small pieces and heat-treated at 610 °C, 630 °C and 650 °C for 2 h, respectively. The obtained glass ceramics were named as GC-610, GC-630 and GC-650, respectively. All the samples were polished for further characterization. The final thickness of PG and glass ceramics is 1.5 mm.

### Characterization

The crystallized phase before and after thermal treatment was identified *via* X-ray diffraction (XRD) measurement (Bruker D2 PHASER Diffractometer), with Cu-K_α_ radiation in 2*θ* range from 10° to 80° with a step length 0.02°. The microstructure of GC-650 was examined on a transmission electron microscope (TEM, Philips-FEI-Tecnai G2 F30). Excitation and emission spectra of Sm^3+^ ions at room temperature were recorded on a fluorescence spectrophotometer equipped with a Hamamatsu R928 photomultiplier tube (Horiba J-Y Fluorolog-3). 450 W xenon lamp was used as the light source. The luminescence decay curves of ^4^G_5/2_ levels of Sm^3+^ ions were recorded on the above apparatus excited at 370 nm. Upconversion luminescence Er^3+^/Yb^3+^ co-doped samples were also performed on the same spectrophotometer and a 980 nm laser diode module (MDL-III-980, China) was used to excite the samples. The output spot size of 980 nm laser was 4 × 6 mm^2^. In order to study the temperature-dependent upconversion luminescence of Er^3+^, the temperature controller (TAP-02) was used to heat the sample from 298 to 573 K and its temperature accuracy is 0.1 K.

## Results and discussion

### Microstructure analysis

**Fig. 1:**
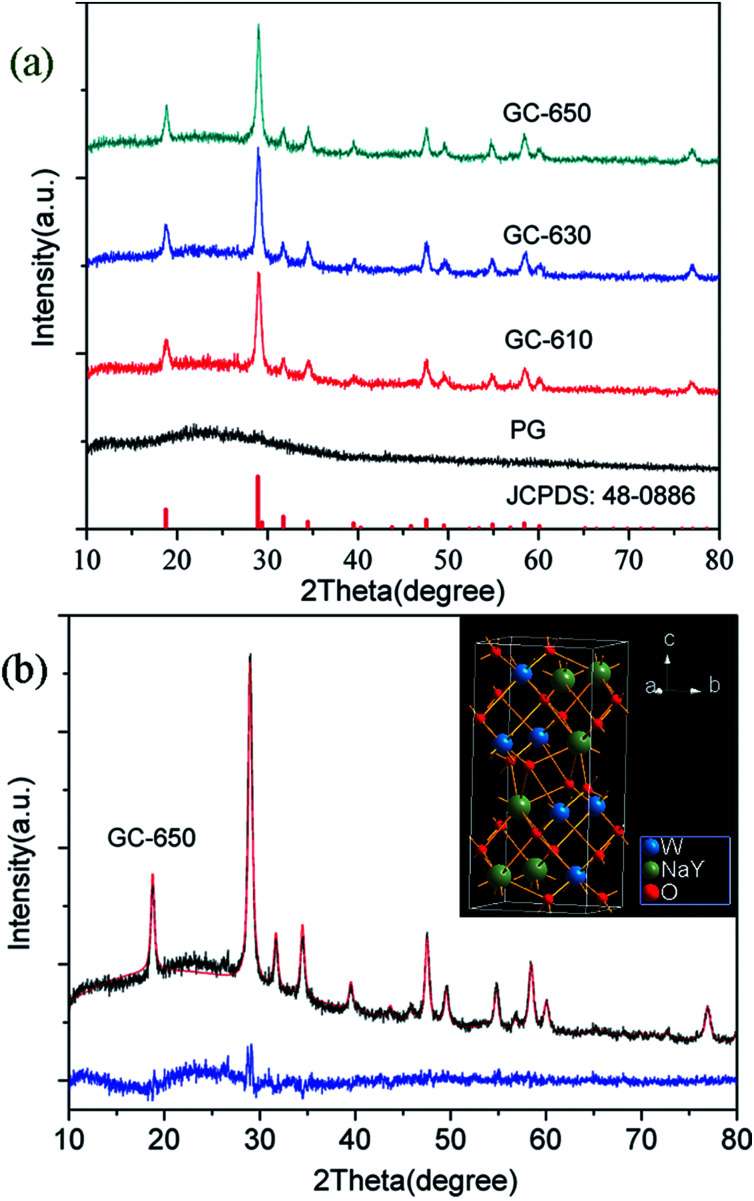
(a) XRD profiles of PG and glass ceramics; (b) Rietveld XRD refinement of GC-650, the inset shows the crystal structure of NaY(WO_4_)_2_.

The XRD profiles of PG and glass ceramics are presented in Fig. 1(a). In the PG, no obvious diffraction peaks are detected in the range of 10–80°, indicative of the amorphous feature of glass matrix. When the samples were thermal-treated at 610, 630 and 650 °C for 2 h, intense diffraction peaks are observed and easily assigned to pure tetragonal NaY(WO_4_)_2_ (JCPDS No 48-0886). The full width at half-maximum (FWHM) of diffraction peaks becomes narrower with the increase of thermal treatment temperature, indicating the gradual growth of NaY(WO_4_)_2_ phase. The average crystalline size of NaY(WO_4_)_2_ nanocrystals could be calculated based on the Scherrer formula,^18^1
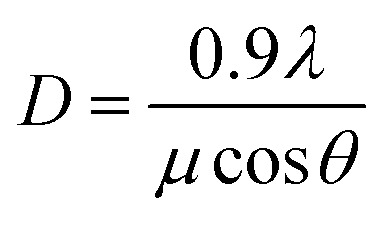
in which *λ* is X-ray wavelength, *μ* is FWHM of diffraction peaks, and *θ* is diffraction angle. The average crystalline sizes were 15.0 nm, 17.8 nm and 20.9 nm for GC-610, GC-630 and GC-650, respectively.

The Rietveld XRD refinement of GC-650 was carried out by using TOPAS software. The initial structural models was NaY_0.95_Yb_0.05_(WO_4_)_2_.^19^Fig. 1(b) show the observed and calculated diffraction peaks as well as the difference of GC-650, which are nearly identical and suggests that GC-650 has a pure tetragonal phase. The fitting reliability parameters of *R*_exp_, *R*_wp_, *R*_p_, and GOF are 3.94, 5.90, 4.82 and 1.5, respectively. The inset of Fig. 1(b) represents the crystal structure of NaY(WO_4_)_2_ drawn by Diamond software. The crystal has a high structural disorder. Especially, Na and Y occupy the same sites in the structure randomly and link with eight oxygen atoms, forming NaO_8_ and YO_8_ edge-sharing polyhedral with S_4_ site symmetry, while W^6+^ ions are coordinated with four oxygen atoms.^20^

**Fig. 2:**
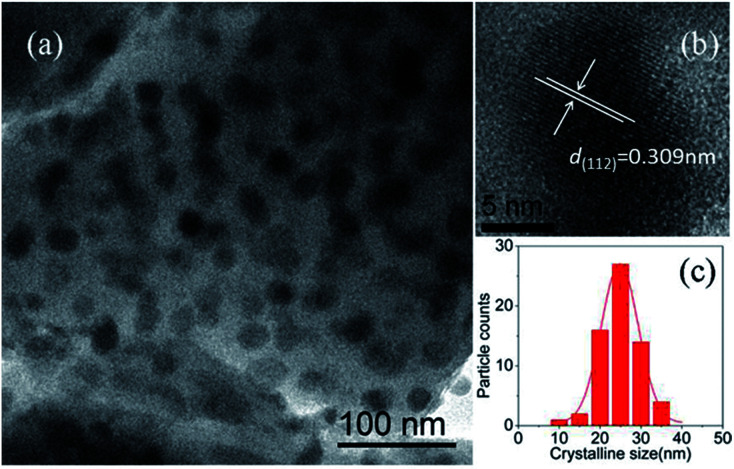
(a) TEM image of NaY(WO_4_)_2_ nanocrystals in the GC-650; (b) HRTEM micrograph of an individual NaY(WO_4_)_2_ nanocrystal; (c) crystalline size distribution of NaY(WO_4_)_2_ nanocrystals in the GC-650.

Fig. 2(a) gives the dark field TEM image of NaY(WO_4_)_2_ nanocrystals in the GC-650. One can observe plenty of dark and nearly spherical nanoparticles, corresponding to the precipitated NaY(WO_4_)_2_ nanocrystals, while the grey background is amorphous glass phase. The HRTEM image of an individual NaY(WO_4_)_2_ nanocrystal shows the well-resolved lattice fringes, which suggests that the precipitated NaY(WO_4_)_2_ nanocrystal possess high crystallinity. The measured interplanar distance is 0.309 nm, which can be well-indexed as the *d*-spacing value of (112) crystal plane of NaY(WO_4_)_2_ crystal (*d*_(112)_ = 0.308 nm). The crystalline size of NaY(WO_4_)_2_ is around 25 nm with a narrow size distribution, which is slightly larger than the value (20.9 nm) calculated from XRD data. The same phenomenon was reported in the PbF_2_ glass ceramics, which was due to the broadening of diffraction peaks and suggested the presence of disorder of the precipitated nanocrystals.^21^

### Photoluminescence and decay lifetimes of Sm^3+^ ions

**Fig. 3:**
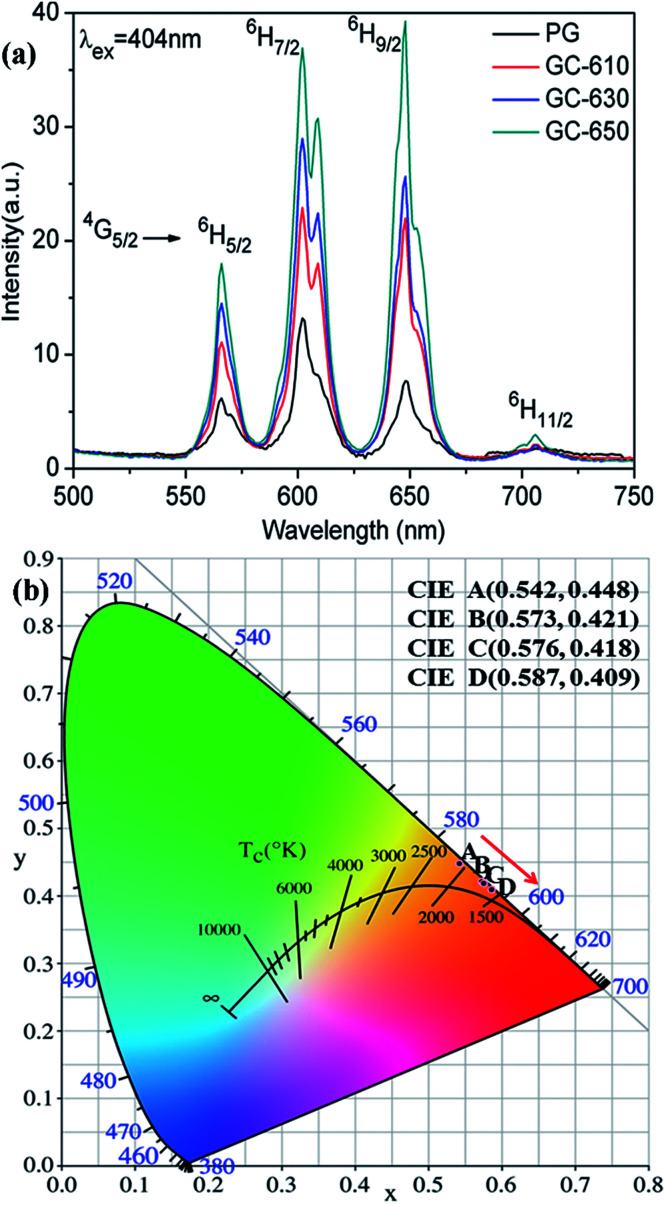
(a) Photoluminescence spectra of Sm^3+^ ions (excited at 404 nm); (b) CIE coordinates of Sm^3+^ doped PG, GC-610, 6C-630 and GC-650, which are labelled as A, B, C, and D, respectively.

Fig. 3 shows the photoluminescence spectra of Sm^3+^ ions in the glass samples before and after thermal treatment. The excitation wavelength is 404 nm. Some strong emission bands at around 566, 602, 648 and 706 nm are observed, which correspond to the transitions from ^4^G_5/2_ energy level to ^6^H_5/2_, ^6^H_7/2_, ^6^H_9/2_ and ^6^H_11/2_ energy levels of Sm^3+^ ions, respectively.^22^ The whole emission intensity of Sm^3+^ ions after thermal treatment is greatly enhanced with the increase of thermal treatment temperature and obvious Stark splitting appear at 602 nm, which is attributed to the segregation of Sm^3+^ ions into the low phonon energy NaY(WO_4_)_2_ nanocrystals by occupying the sites of Y^3+^ ions.^23^ It is notable that the strongest photoluminescence emission band in the PG originates from ^4^G_5/2_ → ^6^H_7/2_ transition at 602 nm, while the strongest photoluminescence emission band in the GC-650 becomes ^4^G_5/2_ → ^6^H_9/2_ transition at 648 nm, which is completely on the contrary to the luminescent behaviour of Sm^3+^ ions in YF_3_ glass ceramics.^24^ Sm^3+^ ions are very sensitive to the subtle change of external environment and acts as a spectroscopic probe to investigate the local site symmetry around active ions in the host matrix. According to the selection rules of electronic transitions,^25^^4^G_5/2_ → ^6^H_5/2_ transition belongs to magnetic dipole transition due to Δ*J* = 0, which is nearly independent of the local environmental change around Sm^3+^ ions, while ^4^G_5/2_ → ^6^H_9/2_ transition is electric transition because of Δ*J* = 2 and which is highly sensitive to external environmental change. In general, the intensity ratio *β* between ^4^G_5/2_ → ^6^H_9/2_ and ^4^G_5/2_ → ^6^H_5/2_ transitions is used to evaluate the local site symmetry around Sm^3+^ ions. When Sm^3+^ ions are situated in the surroundings close to inversion symmetry, ^4^G_5/2_ → ^6^H_5/2_ will be stronger, while in the surroundings far away from inversion symmetry, the ^4^G_5/2_ → ^6^H_9/2_ will be predominant. The calculated *β* values in the PG, GC-610, GC-630 and GC-650 are 1.25, 1.76, 1.98 and 2.18, respectively. The gradual increase of *β* value demonstrates that Sm^3+^ ions are far away from inversion symmetry and enter into a more disordered surrounding after thermal treatment process, which favours the strengthening of photoluminescence intensity for the hypersensitive transitions and is very beneficial to enhance the temperature sensing sensitivity.^26,27^

Due to the relative photoluminescence intensity of Sm^3+^ ions change greatly after thermal treatment, the Commission Internationale de I'Eclairage (CIE) chromaticity coordinates were calculated in order to evaluate the colorimetric performance calculated from the emission spectra. The calculated chromaticity coordinates were A (0.542, 0.448), B (0.573, 0.421), C (0.576, 0.418) and D (0.587, 0.409) for the PG, GC-610, GC-630 and GC-650, respectively, which were labelled in the Fig. 3(b). It is found that the colour changes from yellow orange to reddish orange after thermal treatment, which indicates that glass ceramic doped Sm^3+^ ions could be served as alternative red phosphors in the white LED application.

**Fig. 4:**
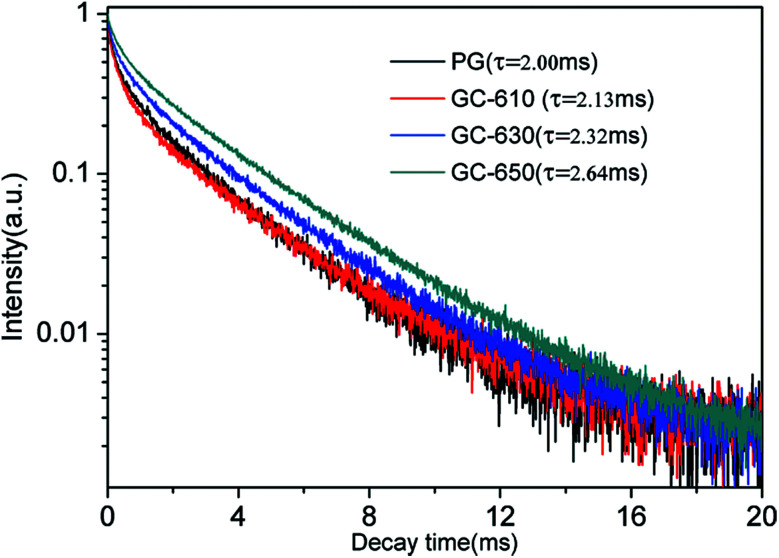
Luminescence decay curves of Sm^3+^ ions in different samples.

The luminescence decay curves of Sm^3+^ ions in different samples are investigated and presented in the Fig. 4. The excitation wavelength is 370 m and the emission wavelength is 602 nm. All the decay curves are well described with a two-exponential equation,^28^2
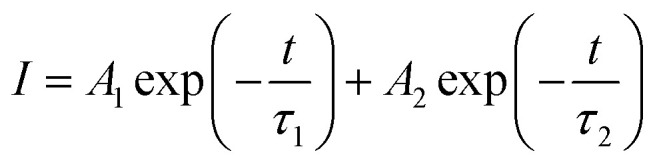
in which *I* is the emission intensity, *A*_1_ and *A*_2_ are the fitting parameters, *τ*_1_ and *τ*_2_ are the slow and rapid lifetimes for exponential components, respectively. The average fluorescence lifetime can be calculated as:3
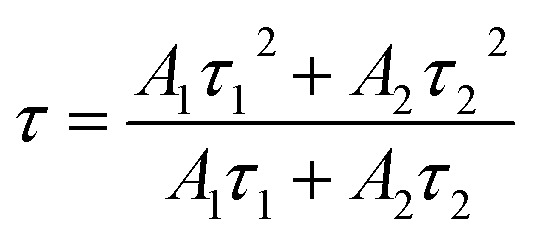


The fitting average fluorescence lifetime of Sm^3+^ ions are 2.00, 2.13, 2.32 and 2.64 ms for PG, GC-610, GC-630 and GC-650, respectively. The gradual increase of fluorescence lifetime of Sm^3+^ ions in the glass ceramics can be ascribed to that much more Sm^3+^ ions partition into the low phonon energy NaY(WO_4_)_2_ nanocrystals and the non-radiative transition rate of Sm^3+^ ions reduces.

### Upconversion luminescence and mechanism of Er^3+^ ions

**Fig. 5:**
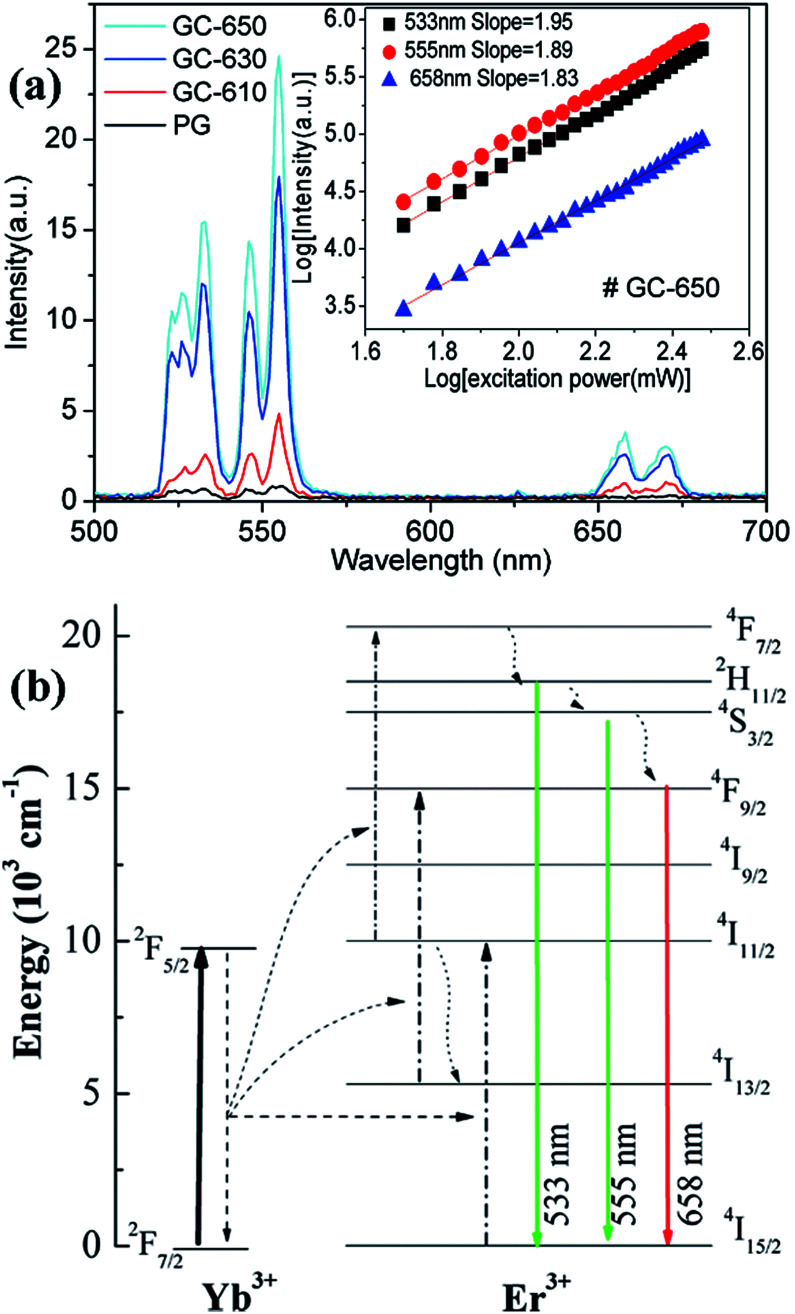
(a) Upconversion emission spectra of Er^3+^/Yb^3+^ ions in different samples under 980 nm excitation; (b) energy level diagram of Er^3+^ and Yb^3+^ and the proposed upconversion luminescence mechanisms Er^3+^ and Yb^3+^.

Fig. 5(a) shows the upconversion emission spectra of Er^3+^/Yb^3+^ ions in different samples at room temperature. The excitation wavelength is 980 nm and its excitation power is 99 mW. Thus, the power density is 0.4125 W cm^−2^, which is enough low and the heating effect caused by 980 nm excitation may be ignored.^29^ Strong green upconversion emission as well as weak red upconversion emission are recorded, which are originated from the ^2^H_11/2_ → ^4^I_15/2_ (533 nm), ^4^S_3/2_ → ^4^I_15/2_ (555 nm) and ^4^F_9/2_ → ^4^I_15/2_ (658 nm) transitions of Er^3+^ ions, respectively. With the increase of thermal treatment temperature, the upconversion intensity is significantly raised and its intensity in GC-650 is 60 times higher than that of PG. The red-to-green emission ratios are 0.263, 0.242, 0.138 and 0.117 for the PG and GC-610, GC-630 and GC-650, respectively. The decrease of red-to-green emission ratio is mainly attributed to the gradual incorporation of Er^3+^ and Yb^3+^ into the low-phonon-energy NaY(WO_4_)_2_ nanocrystals, which effectively reduce the populations of ^4^F_9/2_ energy level through the non-radiative ^4^S_3/2_ → ^4^F_9/2_ transition. Furthermore, intense Stark splitting of Er^3+^ ions due to high crystal field effect also verify the above viewpoint. In order to analyze upconversion mechanism of Er^3+^ ions, the double logarithmic curves of upconversion intensity as a function of excitation power is determined and shown in the inset of Fig. 5(a). For an unsaturated process, the correlation of upconversion intensity *I* and excitation power *P* satisfy the following formula,^30^*I* ∝ *P*^*n*^, in which *n* is the number of the pump photon absorbed in the upconversion process for one visible photon emitted. Thus, the *n* values are equal to the slopes in the inset of Fig. 5(a) and are 1.95, 1.89 and 1.83 for 533 nm, 555 nm, and 658 nm, respectively. These values demonstrate that a double-photon absorption process is responsible for the populations on the ^2^H_11/2_, ^4^S_3/2_ and ^4^F_9/2_ energy levels of Er^3+^ ions. Based on the above data, the upconversion mechanism of Er^3+^, Yb^3+^ ions was proposed and plotted in the Fig. 5(b). Due to its wide absorption cross-section at 980 nm of Yb^3+^ ions, 980 nm light is mainly absorbed by Yb^3+^ and the latter efficiently transfers the energy to the adjacent Er^3+^ ions.^21^ Subsequently, Er^3+^ ions are excited to ^4^F_7/2_ energy level through absorbing double 980 nm photons from Yb^3+^ ions. Then, the ^2^H_11/2_, ^4^S_3/2_ and ^4^F_9/2_ energy levels are successively populated by the rapid non-radiative relaxation from ^4^F_7/2_ energy level, from which green and red upconversion emissions appear. Moreover, the population of ^4^F_9/2_ energy level may be also from the energy transfer process of ^4^I_13/2_ energy level.

### Temperature dependence upconversion luminescence of Er^3+^ ions and temperature sensing performance

**Fig. 6:**
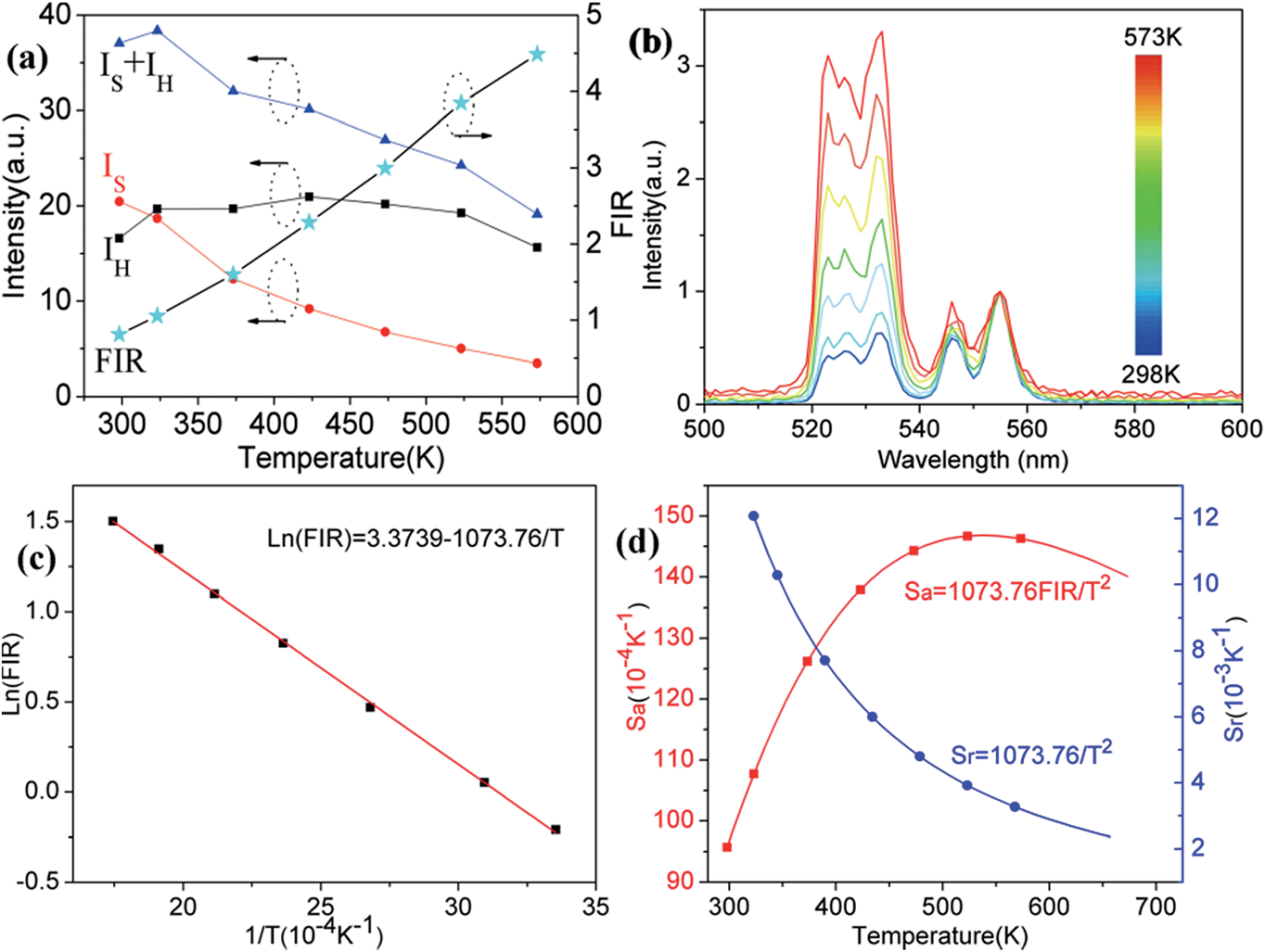
(a) Integrated upconversion intensities of ^2^H_11/2_ (*I*_H_) and ^4^S_3/2_ (*I*_S_) as well as FIR *versus* temperature; (b) normalized upconversion luminescence of Er^3+^ at a temperature range of 298–573 K; (c) ln(FIR) as a function of the inverse absolute temperature 1/*T*; (d) temperature sensitivity *versus* absolute temperature.

In order to investigate the potential of Er^3+^/Yb^3+^ codoped glass ceramics containing NaY(WO_4_)_2_ nanocrystals for optical thermometry based on FIR technique, the dependence of green upconversion luminescence in GC-650 on the temperature was measured at the temperature range from 298 to 573 K and the change of the integrated upconversion intensity of ^2^H_11/2_ (*I*_H_), ^4^S_3/2_ (*I*_S_) as well as ^2^H_11/2_+^4^S_3/2_ (*I*_H_ + *I*_S_) of Er^3+^ ions *versus* temperature is shown in the Fig. 6(a). It is notable that the integrated upconversion intensity *I*_H_ of ^2^H_11/2_ → ^4^I_15/2_ transition first increase and then decrease slowly with the enhancement of environmental temperature. The maximum value of *I*_H_ occurs at 423 K. On the contrary, the integrated upconversion intensity *I*_S_ of ^4^S_3/2_ → ^4^I_15/2_ transition decreases dramatically, which results in the FIR of two energy levels (*I*_H_/*I*_S_) rise from 0.81 to 4.49. In general, when the temperature increases from 298 to 573 K, the upconversion emission become weak due to thermal quenching effect. In our experiment, the whole green upconversion intensity (*I*_H_ + *I*_S_) reduces by 1/2 and intense green emission can be observed at 573 K. This is very beneficial to improve the accuracy of optical data. In order to better reveal the relative change of FIR, the green upconversion emissions were normalized to the intensity at 555 nm and shown in the Fig. 6(b). Clearly, the upconversion emission wavelength changes little, however, the FIR change significantly. Owing to the small bandgap between ^2^H_11/2_ and ^4^S_3/2_, they are thermally coupled and ^2^H_11/2_ energy level may be easily populated *via* thermal excitation from ^4^S_3/2_ energy level and achieve thermal equilibrium, which follows the Boltzmann distribution law. The FIR of upconversion emission is proportional to the ratio of the populations corresponding energy levels and is expressed as:4
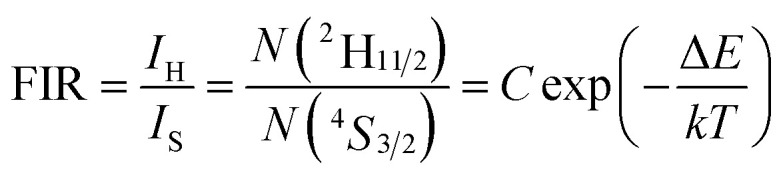
in which *I*_H_ and *I*_S_ are the integrated upconversion intensities for the ^2^H_11/2_ → ^4^I_15/2_ and ^4^S_3/2_ → ^4^I_15/2_ transitions, respectively. The integrated ranges for both transitions are 515–540 nm and 540–570 nm, respectively. *N*(^2^H_11/2_) and *N*(^4^S_3/2_) are the population numbers of ^2^H_11/2_ and ^4^S_3/2_ energy levels, respectively. Δ*E* is the bandgap between the ^2^H_11/2_ and ^4^S_3/2_. *k* is the Boltzmann constant and *T* is the absolute temperature.

The correlation between ln(FIR) and the inverse absolute temperature 1/*T* is described in Fig. 6(c). The linear fitting of the experimental values ln(FIR) as a function of the 1/*T* gives a slope about 1073.76. From this slope, the value of Δ*E* is calculated to be approximately 746.2 cm^−1^, which is very close to the energy gap between the ^2^H_11/2_ and ^4^S_3/2_ levels calculated from the upconversion emission spectra.

The absolute sensitivity (*S*_a_) and relative sensitivity (*S*_r_) are usually used to evaluate the temperature sensing performance and defined as the rate at which the absolute and relative FIR change with the temperature, which could be calculated using the following formula:5
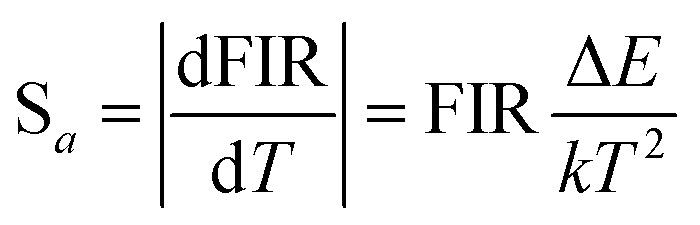
6
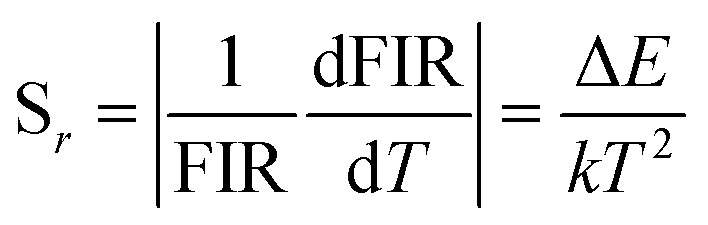


The corresponding sensitivity curves are demonstrated in Fig. 6(d). Table 1 lists the FIR parameters, relative sensitivity *S*_r_, the maximum sensitivity *S*_a_ and the temperatures for the maximum sensitivity in different host matrix. Because *S*_r_ depends only on the energy gap Δ*E*, the *S*_r_ decreases gradually with the increase of temperature. Furthermore, due to its moderate energy gap, *S*_r_ achieved here is comparable to most of values of Er^3+^ ions in other host matrix. The absolute temperature sensitivity *S*_a_ increases rapidly with the increase of temperature and achieves the maximum value 146 × 10^−4^ K^−1^ at 523 K. Clearly, the temperature sensitivity *S*_a_ in our work is much larger than the reported data in fluorides, glass and glass ceramics and slightly larger than the value in tungstates and molybdates. According to Judd–Ofelt theory, S. F. León-Luis proposed that the host matrices with highly distorted local symmetry environments for Er^3+^ ions could obtain large fluorescence intensity ratios and high temperature sensitivities.^16^ Generally, rare earth ions are situated in a symmetric environment in the fluoride, while rare earth ions are located in the asymmetric surroundings in the double tungstates/molybdates due to its highly disordered structure.^41^ The above spectral analysis of Sm^3+^ ions in the NaY(WO_4_)_2_ glass ceramic also confirmed this point. Thus, our experiment provides a new evidence for the viewpoint that the disordered local surroundings around active ions are responsible for the enhancement of the optical sensitivity.

**Table 1:** FIR parameters, relative sensitivity *S*_r_, the maximum sensitivity *S*_a_ and temperatures in different host matrix

Materials	Temperature range (K)	Δ*E* (cm^−1^)	*S*_r_ (%K^−1^)	*S*_a_ (× 10^−4^ K^−1^)	Temperature (K)	Ref.
Er^3+^/Yb^3+^:β-NaYF_4_ phosphor	298–653	706	1015.99/*T*^2^	37	508	12
Er^3+^:oxyfluoride glass	293–720	773	1112/*T*^2^	66	570	16
Er^3+^/Yb^3+^:α-NaYF_4_ glass ceramic	293–720	741	1066/*T*^2^	24	540	16
Er^3+^/Yb^3+^:NaY(WO_4_)_2_ phosphor	133–773	725	1043.12/*T*^2^	112	515	17
Er^3+^/Yb^3+^:NaLa(WO_4_)_2_ phosphor	300–510	719	1005/*T*^2^	131	510	31
Er^3+^/Yb^3+^:Na_0.5_Gd_0.5_MoO_4_ phosphor	298–778	830.4	1195.11/*T*^2^	85.6	590	32
Er^3+^/Yb^3+^:NaBiF_4_ phosphor	248–498	758.1	1094.3/*T*^2^	40	498	33
Er^3+^/Yb^3+^:oxyfluoride glass	291–450	719	1035/*T*^2^	39	513	34
Er^3+^/Yb^3+^:borosilicate glass	293–873	896	1289.09/*T*^2^	31	550	35
Er^3+^/Yb^3+^:K_3_LuF_6_ glass ceramic	300–773	870	1256/*T*^2^	37.6	625	36
Er^3+^/Yb^3+^:β-NaGdF_4_ glass ceramic	303–563	789	1135/*T*^2^	37	580	37
Er^3+^/Yb^3+^:α-NaYF_4_ glass ceramic	298–693	775	1117/*T*^2^	24	560	38
Er^3+^:Sr_2_YbF_7_ glass ceramic	300–500	774	1129.8/*T*^2^	62	560	39
Er^3+^:KYb_2_F_7_ glass ceramic	300–480	844	1224/*T*^2^	45.4	590	40
Er^3+^/Yb^3+^:NaY(WO_4_)_2_ glass ceramic	298–573	746.2	1073.76/*T*^2^	146	523	This work

## Conclusions

An optimized glass composition and an appropriate thermal treatment condition leads to the preparation of a novel glass ceramics, composed of NaY(WO_4_)_2_ nanocrystals dispersed homogeously in the amorphous glass matrix. The microstructure of these nanocrystals were analysed in details based on the XRD and TEM data. The influence of thermal treatment temperature on the luminescence of Sm^3+^ and Er^3+^/Yb^3+^ ions is studied thoroughly. During the devitrification process, Sm^3+^ and Er^3+^/Yb^3+^ ions are gradually segregated into the precipitated low phonon energy NaY(WO_4_)_2_ nanocrystals with a high disorder, which results in the enhancement of photoluminescence emission intensity, well-resolved Stark splitting and long luminescence decay lifetimes. According to the dependence of upconversion intensity on the excitation power, the upconversion mechanism of Er^3+^–Yb^3+^ ions was put forward. The temperature sensing performance was investigated based on the FIR technique and a maximum sensitivity 146 × 10^−4^ K^−1^ was achieved at 523 K. The established correlation of structure, luminescence and temperature sensing provides a new approach to optimize the best host materials for temperature sensing applications.

## Conflicts of interest

There are no conflicts to declare.

## Supplementary Material
